# Single-pulse terahertz spectroscopy monitoring sub-millisecond time dynamics at a rate of 50 kHz

**DOI:** 10.1038/s41467-023-38354-3

**Published:** 2023-05-05

**Authors:** Nicolas Couture, Wei Cui, Markus Lippl, Rachel Ostic, Défi Junior Jubgang Fandio, Eeswar Kumar Yalavarthi, Aswin Vishnuradhan, Angela Gamouras, Nicolas Y. Joly, Jean-Michel Ménard

**Affiliations:** 1 Department of Physics, University of Ottawa, Ottawa, ON K1N 6N5 Canada; 2 Max Planck Centre for Extreme and Quantum Photonics, Ottawa, ON K1N 6N5 Canada; 3 Max Planck Institute for the Science of Light, 91058 Erlangen, Germany; 4 Department of Physics, University of Erlangen-Nürnberg, 91058 Erlangen, Germany; 5 National Research Council Canada, Ottawa, ON K1A 0R6 Canada; 6 Interdisciplinary Center for Nanostructured Films, 91058 Erlangen, Germany

**Keywords:** Terahertz optics, Optical spectroscopy

## Abstract

Slow motion movies allow us to see intricate details of the mechanical dynamics of complex phenomena. If the images in each frame are replaced by terahertz (THz) waves, such movies can monitor low-energy resonances and reveal fast structural or chemical transitions. Here, we combine THz spectroscopy as a non-invasive optical probe with a real-time monitoring technique to demonstrate the ability to resolve non-reproducible phenomena at 50k frames per second, extracting each of the generated THz waveforms every 20 μs. The concept, based on a photonic time-stretch technique to achieve unprecedented data acquisition speeds, is demonstrated by monitoring sub-millisecond dynamics of hot carriers injected in silicon by successive resonant pulses as a saturation density is established. Our experimental configuration will play a crucial role in revealing fast irreversible physical and chemical processes at THz frequencies with microsecond resolution to enable new applications in fundamental research as well as in industry.

## Abstract (web summary)

Single-pulse THz spectroscopy at 50 kHz is performed with a table-top system to extract rapid changes of a material’s complex dielectric function. This technique is demonstrated by monitoring carrier accumulation and relaxation dynamics in silicon.

## Introduction

A large class of phenomena is currently impossible to investigate since they are either too fast, non-reproducible, or both. Slow motion movies and high-speed video captures help us visualize events such as the locomotion of organisms, biological processes, as well as fluid and particle dynamics in the visible and near-infrared (NIR) spectral ranges. Similar time-resolved imaging techniques in the far-infrared could provide unique insight into chemical reactions and physical processes that will not only deepen our understanding of the world around us, but will also reveal insights into future technologies. Water distribution in plants^1^, carrier transport in materials^2^, and protein dynamics^3^ could be analyzed at sub-millisecond timescales, leading to ground-breaking scientific discoveries. In industry, implementation of fast terahertz time-domain spectroscopy (THz-TDS) would provide efficient and non-invasive quality control of goods. For example, in pharmaceutical assembly lines, such a characterization technique would allow companies to determine with great accuracy the material content and thickness of the coating on their tablets, a crucial component of the solid drug delivery mechanism, without sacrificing any satisfactory tablets^4^. The current issue with THz-TDS for these applications, is that the technique mostly relies on electro-optic sampling (EOS) in electro-optic crystals^5,6^. Although EOS in general directly provides the full amplitude and phase information to extract the complex dielectric function of materials, it is a relatively slow point-by-point sampling process that involves mechanically scanning a NIR pulse across a THz wave. To combat this shortcoming, researchers have been attempting to decrease the scanning and data acquisition time required to retrieve the full THz transient by replacing the mechanical delay line by more sophisticated techniques such as rotary mirror arrays^7^, electronically controlled optical sampling^8^, asynchronous optical sampling^9^, optical sampling by cavity tuning^10^, and acousto-optic programmable dispersive filters^11^. These methods of THz detection have increased scanning rates from a fraction of Hz to the kHz range.

Another approach to decreasing data acquisition times in THz-TDS has been to implement single-shot THz detection through time-to-frequency (which includes spectral encoding^12^ and spectral interferometry^13,14^), time-to-space^15^, and time-to-angle^16^ mapping techniques^17^. In fact, THz-TDS of a unique transient state has been realized with these approaches. For example, it has been used to resolve strong magnon–magnon coupling^18^ and measure the complex dielectric function of semiconductors^19^, thin metal films^20^, and other THz materials^21^. Of these listed methods, frequency-to-time mapping by spectral encoding is arguably the most straightforward to implement as it requires only minor modifications to a standard THz-TDS system. It is accomplished by spatially and temporally overlapping a chirped NIR detection pulse with a THz pulse in an electro-optic crystal, such as gallium phosphide (GaP) or zinc telluride (ZnTe), resulting in the time-domain waveform of the THz pulse being imprinted via nonlinear effects onto the NIR spectrum. Single-shot THz detection has been achieved with this technique by resolving THz pulses, generated with ultrafast sources, imprinted onto a NIR pulse with a spectrometer based on cameras^20,22^. Nevertheless, the data collection and data transfer processes performed by the spectrometer can be drastically limited by the speed of the electronics and the large number of photodetectors, which limits the practicality of these approaches. To our knowledge, the fastest table-top shot-to-shot THz-TDS to date is 1 kHz and utilizes the echelon mirror technique^23^. Single-pulse THz detection has also been achieved at MHz acquisition rates using the photonic time-stretch technique^22,24–27^, where the experiments relied on large synchrotron facilities and therefore benefited from large peak power at high repetition rates. Table-top optical sources offer a trade-off between the repetition rate and the peak power, where the latter determines the amplitude of the generated THz electric field. Therefore, a higher repetition rate leads to a lower generated THz field amplitude and a lower signal-to-noise ratio (SNR) in experiments resolving single THz pulses. This trade-off is expected to represent a bottleneck for reaching arbitrarily high repetition rates in single-pulse THz-TDS systems.

Here, we present a complete table-top system capable of single-pulse THz-TDS at a repetition rate of 50 kHz based on chirped-pulse spectral encoding, a photonic time-stretch measurement technique, and fast detection electronics^28,29^. The resulting system relies on a single ultrafast source, which enables the detection of every single generated THz pulse, emitted and detected every 20 μs. To explore the capabilities of our system, we monitor pulse-to-pulse microsecond carrier dynamics in a silicon wafer using successive pairs of NIR pump and THz probe pulses in a transient regime. Each THz wave transmitted through the sample is time-resolved every 20 μs, providing phase and amplitude information, to achieve single-pulse THz spectroscopy of the pump-induced change in the complex dielectric function. With the standard EOS technique, only the equilibrium states of the sample can be measured: the un-pumped or saturated states. With the single-pulse detection technique, we probe the sample at the repetition rate of the laser (50 kHz) to obtain a series of measurements tracing microscopic dynamics that are changing on a pulse-to-pulse basis. Using a theory based on the Drude model, we can extract the density and relaxation time of injected carriers by analyzing the complex transmission spectrum of the THz pulse. We also include in our model dynamic effects such as inhomogeneous carrier distribution in the sample along the THz propagation direction, spatial diffusion, and carrier density-dependent scattering time. The system we present in this work lays the foundation towards the implementation of THz-TDS as a non-invasive tool for quality control of pharmaceuticals and as a low-energy probe to resolve microsecond processes such as the motion of proteins^30,31^, chemical and physical exchange processes^32^, and other complex systems in all fields of scientific research.

## Results

**Fig. 1:**
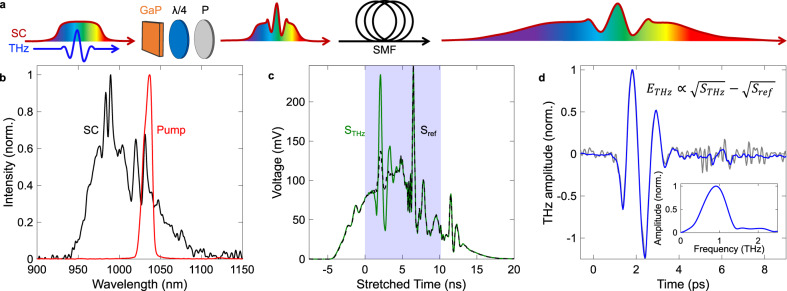
Single-pulse THz detection scheme. **a** Schematic of the spectral encoding of the THz pulse onto the spectrum of the chirped supercontinuum (SC) in a 2-mm-thick gallium phosphide (GaP) crystal. The modulated spectrum is passed through a quarter-wave plate (λ/4) and a polarizer (P) and then coupled into a 2 km-long single-mode fiber (SMF), mapping the frequencies of the NIR pulse to the time-domain to then be resolved on an oscilloscope. **b** Spectrum of the pulse injected into the short polarization maintaining fiber (Pump) and the output supercontinuum (SC), which is used for spectral encoding. **c** Measured signals of the modulated (*S*_THz_) and unmodulated (*S*_ref_) spectra on the oscilloscope. The area highlighted in blue represents the relevant section of the spectrum which contains the THz transient. **d** Extracted THz electric field (*E*_THz_) from the curves in **c** and the Fourier transform of the averaged transient (inset). The blue line is the signal averaged over 10k pulses while the gray line is a single-pulse measurement.

Experiments are performed with an amplified ultrafast laser source delivering femtosecond pulses at a 50 kHz repetition rate. We generate THz transients by optical rectification using the standard tilted-pulse-front configuration in a lithium niobate (LiNbO_3_) crystal^33^ and resolve each transient with a structured NIR gating pulse as schematically shown in Fig. 1a. This gating pulse is obtained by launching a NIR pulse into a 2 meter-long polarization-maintaining fiber (PMF). Self-phase modulation and linear dispersion in the fiber yield a chirped NIR supercontinuum (SC) with ~100 nm bandwidth and 6 ps pulse duration. The spectra measured before and after nonlinear propagation in the PMF are shown in Fig. 1b. The stretched pulse is then used to encode, through a nonlinear interaction process, an oscillating THz transient by spatially and temporally overlapping the two beams into a second-order nonlinear crystal. After the nonlinear interaction process, the oscillating THz field is encoded in the polarization state of individual spectral components of the SC. To read this information with optimal sensitivity, we first use a quarter-wave plate and a linear polarizer to efficiently extract the THz-modulated signal while blocking most of the unaltered NIR beam. We then perform the photonic time-stretch technique, a single-pulse spectroscopy technique also known as the dispersive Fourier transform, by dispersing spectral components of the polarization-filtered SC into a 2 km-long commercial single-mode fiber (SMF) before time-resolving each nanosecond-stretched NIR pulse with fast electronics. A background signal is removed by subtracting the unmodulated SC (*S*_ref_ in Fig. 1c) from the THz-modulated SC (*S*_THz_ in Fig. 1c). Both signals, approximately 20 ns in duration, recorded with the oscilloscope after propagation through the 2 km-long SMF are plotted in Fig. 1c. The resulting signal is the THz transient (*E*_THz_), which is shown in Fig. 1d along with its spectrum (inset) corresponding to the Fourier transform. The derivation of the formula shown in Fig. 1d can be found in section 2 of the Supplementary Information. The oscilloscope time axis is calibrated by comparing measurements obtained at different time intervals between the THz and detection NIR pulses, which is accurately controlled by a delay stage^34^. The resulting linear relationship between relative delay (in ps) and stretched time on the oscilloscope (in ns) yields the calibration factor, otherwise known as the time-stretch factor, of 1130. With this technique, the THz acquisition rate is determined by the repetition rate of the laser; meaning that with a laser emitting NIR pulses at a rate of 50 kHz, we retrieve corresponding THz waveforms every 20 μs.

### Monitoring pulse-to-pulse carrier accumulation dynamics

We demonstrate the capabilities of our single-pulse THz detection scheme by performing optical-pump THz-probe spectroscopy on 0.9 mm-thick nominally undoped silicon to monitor carrier dynamics. Such measurements can notably be used to monitor the accumulation of optically injected carriers from successive pump pulses or the gradual recombination of free carriers. Intrinsic silicon has a relatively long carrier relaxation time of a few hundred microseconds at low carrier densities. As a result, successive resonant pump pulses at a repetition rate of 50 kHz lead to carrier accumulation until a saturation density is reached. In general, the single-pulse technique provides a unique access to fast dynamical systems as standard pump-probe schemes are not only inadequate to probe non-reproducible phenomena, but would require km-long delay lines to map out microsecond dynamics.

**Fig. 2:**
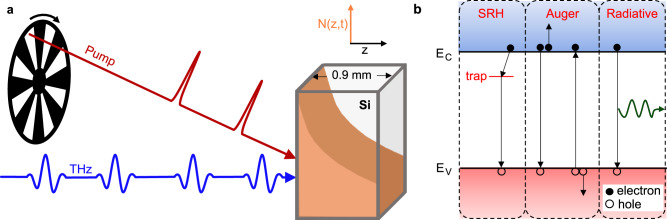
Optical-pump THz-probe single-pulse THz spectroscopy. **a** Schematic of the experimental configuration used to measure the low-energy response of optically injected carrier density *N*(*z*, *t*) (orange) in a 0.9 mm-thick nominally undoped silicon (Si) sample. The train of NIR resonant pulses (Pump) can be chopped to investigate the dynamics of pulse-to-pulse carrier injection followed by carrier relaxation. **b** Schematic of the recombination mechanisms considered in simulations where the red area represents the valence band (*E*_V_), and blue represents the conduction band (*E*_C_). Shockley–Read–Hall (SRH), Auger, and radiative recombination mechanisms are considered.

We perform these experiments in two manners: (i) by activating the pump and probe pulses simultaneously to observe the carrier saturation dynamics and (ii) by inserting a chopper in the pump beam to monitor both the carrier accumulation and recombination dynamics, which are cyclically reproduced as the pump beam is being blocked and unblocked (Fig. 2a). We use a translation stage to adjust the time delay between the THz probe and NIR pump pulses, allowing us to adjust the THz probe to any time delay before or after carrier injection by the corresponding NIR pump pulse. When the probe shortly precedes the pump pulse, spectroscopy measurements become sensitive to residual carriers with unique characteristics as they undergo about 20 µs (or the inverse of the laser repetition rate) of thermalization, diffusion and recombination. Inhomogeneous carrier distribution (*N*(*z,t*)) along the propagation direction of the pump (*z*) is assumed as well as Shockley–Read–Hall (SRH), Auger, and radiative carrier recombination mechanisms (Fig. 2b). We also consider spatial diffusion in our model.

**Fig. 3:**
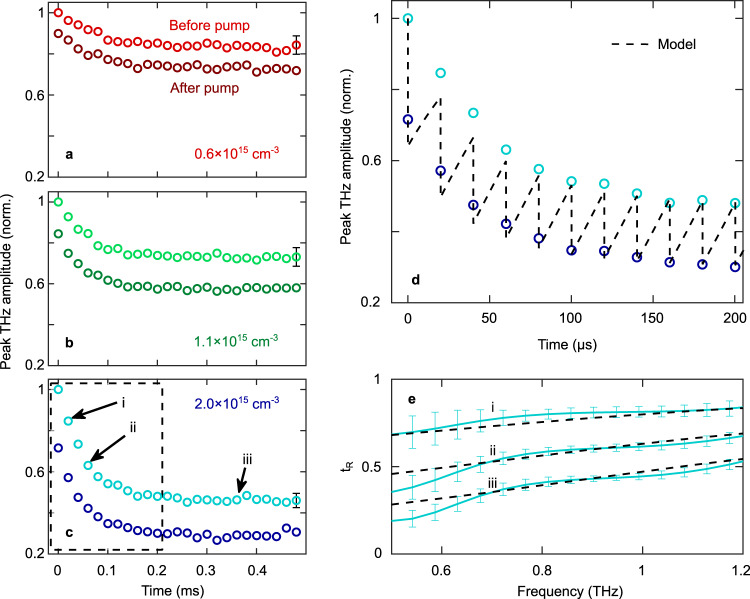
Carrier accumulation dynamics in silicon monitored with single-pulse THz detection. The peak of the THz transient as the pump pulses arrive before (dark circles) and after (light circles) the THz pulse for carrier densities of **a** 0.6 (red), **b** 1 (green), and **c** 2 (blue) $\times$10^15 ^cm^−3^. The area inside the dashed line box in **c** is displayed in **d** along with the results from theoretical calculations (dashed black line). **e** Transmission of the amplitude spectra relative to the unpumped silicon (*t*_R_) of the pulses labeled *i*–*iii* in Fig. 3c and results from the theoretical model (dashed black line). Theoretical time dynamics are calculated with the Drude model and the free carrier rate equation considering an initial recombination time of 30 µs, a density of available traps of 6$\times$10^11^ cm^−3^, and an initial scattering time of 190 fs. Error bars throughout all panels represent the standard deviation over ten measurements.

Figures 3a–c show the peak amplitude of the transmitted THz pulse, which can be used to indicate the carrier density as a function of time. We explore three pump power regimes injecting different carrier densities corresponding to 0.6$\,\times \,$10^15^, 1.1$\,\times \,$10^15^, and 2$\,\times \,$10^15^ cm^-3^ per pulse. The experiments are repeated several times for each carrier density considered in order to extract a standard deviation – the data presented in Fig. 3 is averaged over ten iterations. Obtaining a standard deviation is an essential step in evaluating the signal-to-noise ratio. The fact that the silicon wafer experiences the same equilibrium state before optical pumping allows us to perform several experiments under the same conditions. The standard deviation, represented as error bars in Fig. 3a–c, quantitatively demonstrates the reproducibility and validity of our single-pulse THz-TDS technique and shows a relative variation of ~10% for all data points. The experiment is performed as the THz probe pulse is delayed by 300 ps after the NIR pump pulse (dark colored circles) or precedes the same pump pulse by 130 ps (light colored circles). In our experiment, the saturation carrier density is reached after 200 µs, corresponding to 10 pump pulses. At low pump pulse energy, fewer carriers are injected into the sample resulting in a lower absorption of the THz field and a higher overall transmission amplitude. Conversely, the transmission is the lowest at the highest pump pulse energy. Here, we take advantage of the fact that optically injected carrier dynamics in semiconductors is a pulse-to-pulse reproducible process to average data collected over ten measurements. In Fig. 3d, we plot the same data as in Fig. 3c on a shorter time scale to resolve fine details of the time-varying THz transmission during carrier accumulation from the successive NIR pump pulses. As carriers accumulate in the sample with each pump pulse, we observe an increase of the rate of carrier recombination. Our experimental results agree well with numerical calculations of photocarrier dynamics in silicon (dashed black line). In these simulations, we model the inhomogeneous injected carrier distribution by considering multiple thin slices of fixed carrier concentration across the Si sample in the direction of THz propagation (*z*). Carrier relaxation mechanisms listed in Fig. 2b are considered for each slice as well as carrier diffusion across neighboring slices. The THz absorption of each region is modeled with the Drude model and Beer–Lambert law. Then, these results are combined to obtain the transmission amplitude through the whole sample thickness. Calculations are carried out with an initial trap-assisted effective recombination time of 30 µs, a density of available traps of 6$\times$10^11 ^cm^-3^ and an intrinsic scattering time of 190 fs. With these standard parameters^35–37^, our model and experimental measurements are in good agreement. A more detailed description of the model is provided in section 3 of the Supplementary Information. The real part of the transmitted THz amplitude spectrum relative to the unpumped silicon (*t*_R_) of three measurements labeled *i*–*iii* in Fig. 3c is displayed in Fig. 3e, error bars corresponding to the standard deviation over ten measurements, along with the corresponding calculated transmission spectra using the model described above (dashed black lines). Here we limit our analysis to the frequency components contained within the FWHM of the THz spectrum shown in Fig. 1d. We also find a good agreement between the model and the measurements in this case. Since carrier-induced absorption preferentially reduces the spectral amplitude at low frequencies, creating a steep spectral edge, we obtain the smallest error bars in the region around 0.9 THz and slightly higher ones near 0.6 THz. Note that an experimental THz scheme relying on the standard EOS detection technique could be used to characterize steady states, corresponding here to the measurements *iii* in the saturation regime, but not the microsecond dynamics revealing transient states, such as the measurements corresponding to *i* and *ii*.

**Fig. 4:**
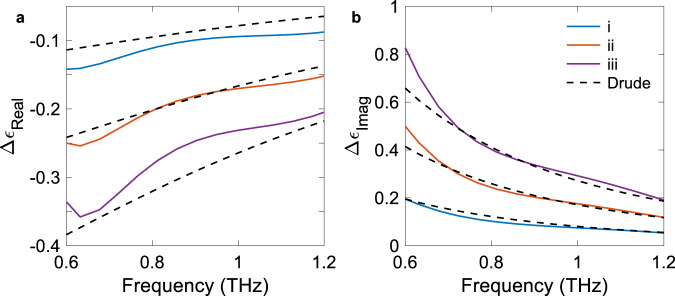
Pulse-to-pulse changes in the complex dielectric function of optically pumped silicon. Extracted **a** real and **b** imaginary change in dielectric function calculated from the transmission spectra presented in Fig. 3e (pulses labeled *i*, *ii*, and *iii*) and the phase information of the THz pulses. Carrier distribution and scattering time (fixed at 155 fs) are used as fitting parameters for the Drude model.

We use the complex transmission amplitude to calculate the change in the real and imaginary parts of the dielectric function, which are shown in Fig. 4a, b, respectively^38^. In our experiment, we can neglect the effect of the carrier density on the Fresnel transmission coefficient. As a result, the phase difference between the pumped and unpumped silicon is associated with the change in the refractive index of the sample, while *t*_*R*_, shown in Fig. 3e, is directly associated to the change in absorption. We consider the same three measurements *i*–*iii*, as those identified in Fig. 3c, e, but this time we overlay the experimental results with a standard Drude model. The basic model is based on a homogenous carrier distribution, which is a valid approximation considering that diffusion has 20 µs after the last NIR excitation pulse to flatten the carrier distribution. We also set a non-carrier dependent scattering time for simplicity. Overall, we observe excellent agreement (dashed black lines) with carrier density corresponding to 4$\times$10^14^, 8.5$\times$10^14^, 1.4$\times$10^15 ^cm^−3^ and a scattering time corresponding to 155 fs. The data presented in Figs. 3 and 4 shows that our single-pulse THz detection scheme can reliably perform spectroscopy and extract material parameters at a rate of 50 kHz, allowing us to observe dynamical changes in a system by resolving each successive THz pulse separated by 20 µs. Standard pump-probe techniques intrinsically relying on pulse averaging cannot be used to directly extract the same information.

### Resolving carrier accumulation and relaxation

**Fig. 5:**
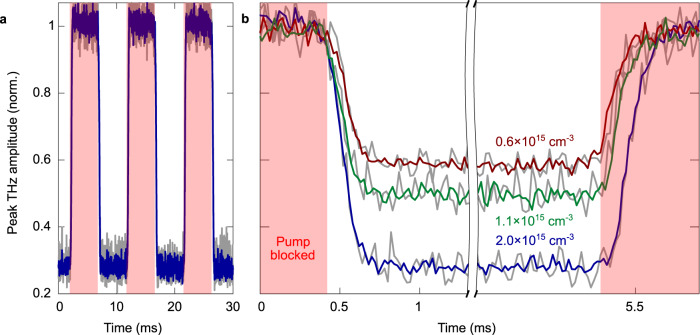
Amplitude variations of THz transients tracing cyclical carrier accumulation and relaxation dynamics. Optical-pump THz-probe results achieved by tightly focusing the pump beam into a mechanical optical chopper, with the pump pulse arriving before the THz probe. **a** Injected carrier density is fixed at 2$\times$10^15 ^cm^-3^ and the peak THz amplitude from single-pulse THz detection is extracted over three chopper cycles. **b** Peak THz amplitude extracted the same way as in **a**, with carrier densities of 0.6 (red), 1.1 (green), and 2 (blue) $\times$10^15^ cm^-3^. The areas highlighted in red correspond to the situation without any pump. Colored lines represent data averaged over 3 chopper cycles, while gray lines are single-pulse measurements.

In a second type of experiment, we use a chopper wheel to block and unblock the pump beam. Carrier accumulation and recombination dynamics in Si are recorded cyclically at a frequency of 100 Hz while measuring the transmitted THz transient with the single-pulse detection technique. Figure 5a shows the peak THz amplitude over three chopper cycles and Fig. 5b provides a closer look over a single chopper cycle. This configuration not only allows us to measure the carrier injection dynamics as in Fig. 3, but also passively measures the carrier recombination dynamics when the pump pulse is blocked by the chopper (red highlighted area). On the left side of Fig. 5b, after the pump is unblocked, we have the carrier injection rate for each previously considered pump energies; and on the right side of Fig. 5b, after the pump is blocked, we have carrier recombination times which depend on the carrier density in the silicon. Our experiment yields the full recombination dynamics occurring over a millisecond time scale, while standard optical-pump THz-probe systems relying on a translation stage to incrementally change the time delay between the pump and probe are limited to nanoseconds scanning range due to the physical size of the stage. More importantly, the THz time-domain signal is sampled at a frequency of 50 kHz, corresponding to the repetition rate of the laser, allowing us to resolve fast and complex temporal dynamics. In our experiment, the excitation pulse is periodically chopped, allowing measurements to be repeated over several identical cycles. As shown in Fig. 5b, the SNR corresponding to single-pulse (gray lines) data is increased when we average over three cycles (colored lines) because carrier dynamics in semiconductors are reproducible for identical optical excitation conditions. However, no averaging is intrinsically required to monitor low-energy dynamics, and this is one of the main advantages of this technique. As such, we believe our system to be appropriate for the exploration of non-reproducible phenomena such as oxidation or combustion, or the study of chaotic dynamical systems notably arising in chemistry and biology. Finally, we believe improvements to our current setup will lead to an improved SNR of single-pulse measurements allowing us to operate the system at MHz frequencies to resolve sub-microsecond dynamics.

In summary, we have presented a table-top system capable of single-pulse THz spectroscopy by combining chirped-pulse spectral encoding and a photonic time-stretch. With this technique, we have experimentally revealed pulse-to-pulse carrier dynamics and changes in the complex dielectric function of silicon. We have validated these experimental results with theory considering diffusion and Shockley-Read-Hall, Auger, and radiative recombination mechanisms. Although we performed our THz spectroscopy experiments at a repetition rate of 50 kHz, revealing sub-millisecond dynamics in silicon, our acquisition rate was limited only by the signal-to-noise ratio at higher repetition rates. Further development of this system will enable us to reach acquisition rates in the MHz to reveal sub-microsecond processes in systems resonant to THz frequencies. This THz-TDS technique promises to be a powerful tool for the observation of fast physical, biological, and chemical processes, non-reproducible phenomena, chaotic systems, and a robust technique for rapid non-invasive quality control in industrial applications.

## Methods

The setup relies on an amplified ultrafast laser source delivering 180 fs pulses (FWHM) at a central wavelength of 1030 nm, a pulse energy of 120 μJ, and a repetition rate of 50 kHz. The optical beam is split into three paths (a schematic of the experimental setup is shown in Fig. S1 of the Supplementary Information). In the first path, NIR pump pulses impinge on the silicon sample for resonant excitation. In the second path, where most of the optical power lies, the NIR pulses are used to generate THz pulses in a lithium niobate (LiNbO_3_) crystal with a tilted-pulse-front configuration^33^. In the third path, the NIR beam is launched into a 2 m-long PMF (OZ Optics PMF-980-6/125-0.25-L) to obtain a chirped SC with ~100 nm bandwidth and 6 ps pulse duration. This stretched pulse is then used to encode, through a nonlinear interaction process in a 2 mm-thick 110-oriented GaP crystal, an oscillating THz transient. Using this technique, we can trace an oscillating THz field with a resolution of δt = 300 fs (see section 1 of the Supplementary Information for details about this calculation)^12^, reliably resolving frequencies up to 1.6 THz according to the Shannon–Nyquist criteria. Polarization filtering is performed with a quarter-wave plate and a linear polarizer to limit the transmission of unaltered NIR light. We then perform the photonic time-stretch technique by dispersing spectral components of the polarization-filtered SC into a 2 km-long SMF (Corning HI1060 flex) before time-resolving each nanosecond-stretched NIR pulse with a fast photodiode (Newport 1544-B, 12 GHz, 32 ps rise time) and a GHz-bandwidth oscilloscope (Tektronix MSO68B, 10 GHz).

### Reporting summary

Further information on research design is available in the Nature Portfolio Reporting Summary linked to this article.

## Supplementary information

Supplementary Information

Reporting Summary

## Data Availability

The data in this study have been deposited in the Figshare database and are available to the public at 10.6084/m9.figshare.22575004.

## Code availability

The code used in this study is available from the corresponding author upon request.

## References

[1] Shchepetilnikov AV, Zarezin AM, Muravev VM, Gusikhin PA, Kukushkin IV (2020). Quantitative analysis of water content and distribution in plants using terahertz imaging. Opt. Eng..

[2] Delport G, Macpherson S, Stranks SD (2020). Imaging carrier transport properties in halide perovskites using time-resolved optical microscopy. Adv. Energy Mater..

[3] Wolff, A. M. et al. Mapping protein dynamics at high-resolution with temperature-jump X-ray crystallography. Preprint at *bioRxiv*10.1101/2022.06.10.495662 (2022).

[4] Ho L (2007). Analysis of sustained-release tablet film coats using terahertz pulsed imaging. J. Controlled Release.

[5] Aoki K, Savolainen J, Havenith M (2017). Broadband terahertz pulse generation by optical rectification in GaP crystals. Appl. Phys. Lett..

[6] Leitenstorfer A, Hunsche S, Shah J, Nuss MC, Knox WH (1999). Detectors and sources for ultrabroadband electro-optic sampling: experiment and theory. Appl. Phys. Lett..

[7] Molter D (2010). High-speed terahertz time-domain spectroscopy of cyclotron resonance in pulsed magnetic field. Opt. Express.

[8] Kim Y, Yee D-S (2010). High-speed terahertz time-domain spectroscopy based on electronically controlled optical sampling. Opt. Lett..

[9] Yasui T, Saneyoshi E, Araki T (2005). Asynchronous optical sampling terahertz time-domain spectroscopy for ultrahigh spectral resolution and rapid data acquisition. Appl. Phys. Lett..

[10] Hochrein T (2010). Optical sampling by laser cavity tuning. Opt. Express.

[11] Urbanek B (2016). Femtosecond terahertz time-domain spectroscopy at 36 kHz scan rate using an acousto-optic delay. Appl. Phys. Lett..

[12] Jiang Z, Zhang X-C (1998). Electro-optic measurement of THz field pulses with a chirped optical beam. Appl. Phys. Lett..

[13] Sharma G, Singh K, Al-Naib I, Morandotti R, Ozaki T (2012). Terahertz detection using spectral domain interferometry. Opt. Lett..

[14] Zheng S (2018). Improved common-path spectral interferometer for single-shot terahertz detection. Photonics Res..

[15] Shan J (2000). Single-shot measurement of terahertz electromagnetic pulses by use of electro-optic sampling. Opt. Lett..

[16] Minami Y, Hayashi Y, Takeda J, Katayama I (2013). Single-shot measurement of a terahertz electric-field waveform using a reflective echelon mirror. Appl. Phys. Lett..

[17] Teo SM, Ofori-Okai BK, Werley CA, Nelson KA (2015). Invited Article: Single-shot THz detection techniques optimized for multidimensional THz spectroscopy. Rev. Sci. Instrum..

[18] Makihara T (2021). Ultrastrong magnon–magnon coupling dominated by antiresonant interactions. Nat. Commun..

[19] Minami Y, Horiuchi K, Masuda K, Takeda J, Katayama I (2015). Terahertz dielectric response of photoexcited carriers in Si revealed via single-shot optical-pump and terahertz-probe spectroscopy. Appl. Phys. Lett..

[20] Russell BK (2017). Self-referenced single-shot THz detection. Opt. Express.

[21] Du L (2021). Organic crystal-based THz source for complex refractive index measurements of window materials using single-shot THz spectroscopy. Appl. Phys. A.

[22] Roussel E (2022). Phase diversity electro-optic sampling: a new approach to single-shot terahertz waveform recording. Light Sci. Appl..

[23] Gao FY, Zhang Z, Liu Z-J, Nelson KA (2022). High-speed two-dimensional terahertz spectroscopy with echelon-based shot-to-shot balanced detection. Opt. Lett..

[24] Roussel E (2015). Observing microscopic structures of a relativistic object using a time-stretch strategy. Sci. Rep..

[25] Evain C (2017). Direct observation of spatiotemporal dynamics of short electron bunches in storage rings. Phys. Rev. Lett..

[26] Szwaj C (2016). High sensitivity photonic time-stretch electro-optic sampling of terahertz pulses. Rev. Sci. Instrum..

[27] Steffen B (2020). Compact single-shot electro-optic detection system for THz pulses with femtosecond time resolution at MHz repetition rates. Rev. Sci. Instrum..

[28] Tong YC, Chan LY, Tsang HK (1997). Fibre dispersion or pulse spectrum measurement using a sampling oscilloscope. Electron. Lett..

[29] Goda K, Solli DR, Tsia KK, Jalali B (2009). Theory of amplified dispersive Fourier transformation. Phys. Rev. A.

[30] Otosu T, Ishii K, Tahara T (2015). Microsecond protein dynamics observed at the single-molecule level. Nat. Commun..

[31] Thompson MC (2019). Temperature-jump solution X-ray scattering reveals distinct motions in a dynamic enzyme. Nat. Chem..

[32] Dzikovski B (2020). Microsecond exchange processes studied by two-dimensional ESR at 95 GHz. J. Am. Chem. Soc..

[33] Hebling J, Yeh K-L, Hoffmann MC, Bartal B, Nelson KA (2008). Generation of high-power terahertz pulses by tilted-pulse-front excitation and their application possibilities. J. Opt. Soc. Am. B.

[34] Kobayashi M (2016). High-acquisition-rate single-shot pump-probe measurements using time-stretching method. Sci. Rep..

[35] Schroder DK (1997). Carrier lifetimes in silicon. IEEE Trans. Electron Devices.

[36] Yokoyama K, Lord JS, Miao J, Murahari P, Drew AJ (2017). Photoexcited muon spin spectroscopy: a new method for measuring excess carrier lifetime in bulk silicon. Phys. Rev. Lett..

[37] Jacoboni C, Canali C, Ottaviani G, Alberigi Quaranta A (1977). A review of some charge transport properties of silicon. Solid State Electron..

[38] Withayachumnankul W, Naftaly M (2014). Fundamentals of measurement in terahertz time-domain spectroscopy. J. Infrared Millim. Terahertz Waves.

