# Supplementary material for: Single-pulse terahertz spectroscopy monitoring sub-millisecond time dynamics at a rate of 50 kHz
Source: Nat Commun. 2023 May 5;14:2595. doi: 10.1038/s41467-023-38354-3 (PMC10163249; doi:10.1038/s41467-023-38354-3)
Supplement: Supplementary file 1 — Supplementary Information [file 41467_2023_38354_MOESM1_ESM.pdf]

Supplementary Information: Single-pulse terahertz spectroscopy monitoring sub-millisecond time dynamics at a rate of 50 kHz

Nicolas Couture[1,2,*], Wei Cui[1,2], Markus Lippl[3,4], Rachel Ostic[1,2], Défi Junior Jubgang Fandio[1,2], Eeswar Kumar Yalavarthi[1,2], Aswin Vishnuradhan[1,2], Angela Gamouras[1,5], Nicolas Y. Joly[4,3,6], and Jean-Michel Ménard[1,2,5,**]

[1]Department of Physics, University of Ottawa, Ottawa, Ontario K1N 6N5, Canada
[2]Max Planck Centre for Extreme and Quantum Photonics, Ottawa, Ontario K1N 6N5, Canada
[3]Max Planck Institute for the Science of Light, Erlangen 91058, Germany
[4]Department of Physics, University of Erlangen-Nürnberg, Erlangen 91058, Germany
[5]National Research Council Canada, Ottawa, Ontario K1A 0R6, Canada
[6]Interdisciplinary Center for Nanostructured Films, Erlangen 91058, Germany
[*]Corresponding author: ncout007@uottawa.ca
[**]Corresponding author: jean-michel.menard@uottawa.ca

1. Full description and schematic of the optical-pump THz-probe experimental setup

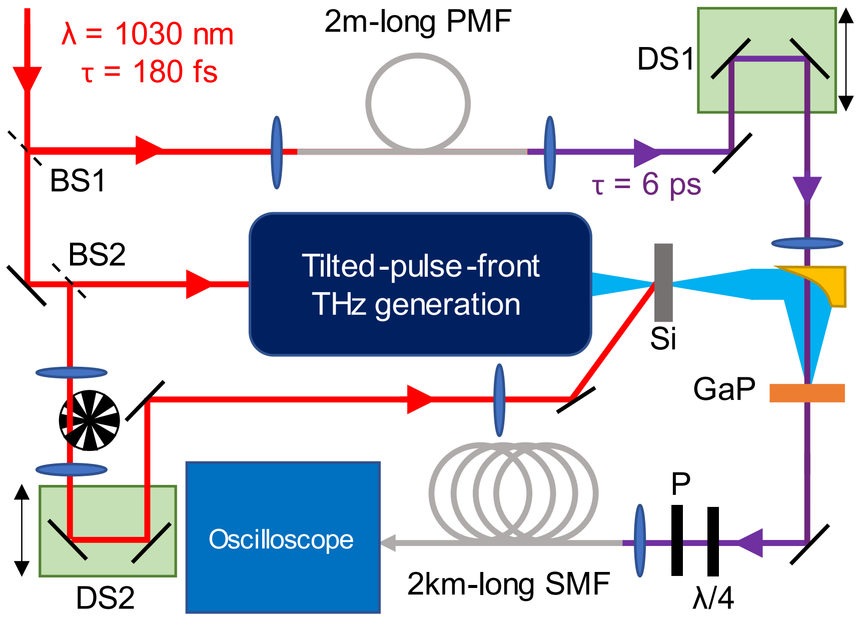

Figure S1. Schematic of the optical-pump THz-probe experimental setup. BS: beamsplitter; PMF: polarization maintaining fiber; DS: delay stage; Si: silicon wafer; GaP: Gallium Phosphide; λ/4: quarter-wave plate; P: polarizer; SMF: single-mode fiber.

The output from a Yb:KGW ultrafast source (1030 nm central wavelength, 120 μJ pulse energy, 50 kHz repetition rate, 180 fs FWHM time duration) (red line) is split into two initial paths with a beamsplitter (BS1, 10R:90T). The reflected beam (12 μJ), to be used as a detection pulse, is attenuated to ~10 nJ and launched into a 2-meter-long polarization maintaining fiber (PMF) whose slow axis is oriented parallel to the linear polarization of the incoming pulse. Self-phase modulation and linear dispersion in the fiber yield a NIR supercontinuum (SC) with a spectral bandwidth approaching ~100 nm and a time duration of 6 ps (purple line Fig. S1). The temporal resolution of this method is given by $\delta t = \sqrt{T_0 T_C}$, where T_0 is the unchirped time duration of the SC in the Fourier transform limited case and T_C is the time duration of the chirped pulse[1]. Although the spectral profile of the SC is not Gaussian (Fig. 1b of the main manuscript), a spectral bandwidth of ~100 nm (at 10 dB) is available for spectral encoding; meaning T_0 ~15 fs when assuming a Gaussian temporal profile. The result is a temporal resolution δt = 300 fs, which is sufficient to

resolve the bandwidth of our THz pulse according to the Shannon-Nyquist criterion. The remaining power is passed through a second beamsplitter (BS2, 20R:80T); most of the energy (~86 μJ) is used for THz generation via the tilted-pulse-front technique[2] and the remainder (~22 μJ) is used to optically pump the 0.9-mm-thick silicon wafer (Si). The pump beam is attenuated with neutral density filters (not shown in Fig. S1) to achieve the various carrier injection rates shown in Fig. 3 of the main manuscript. An optical chopper is placed in the beam pumping the silicon, placed at the focus of a 1x telescope. The chopper blade spins fast enough such that fewer than five pulses are only partially transmitted (on average). Delay stages adjust the temporal delay between the detection and THz pulses (DS1), and between the pump and THz pulses (DS2). The experimental setup is used in one of two manners: i) the chopper is off and the pulsed laser output is actively controlled with the pulse picker of the laser; and ii) the laser remains operational with a continuous pulse train output and the chopper is turned on to activate and deactivate the pump at a frequency of 100 Hz. The spot size of the NIR pump on the Si wafer has a radius of ~2 mm at $1/e^2$. The THz pulse transmitted through the Si wafer and the NIR detection pulse are overlapped in a 2-mm-thick gallium phosphide (GaP) crystal. The time domain THz waveform modulates the spectrum of the NIR detection pulse, passes through a quarter-wave plate (λ/4) and polarizer (P), and is launched into a 2-km-long single-mode fiber (SMF). The quarter-wave plate, with its fast axis aligned to the polarization of the unmodulated chirped SC, and linear polarizer, oriented such that the unmodulated chirped SC is nearly completely attenuated (~97% attenuation), filter out unmodulated NIR background light reaching the detector, thus increasing the signal-to-noise ratio of the measurement[1]. The SMF stretches the THz-modulated NIR pulse to the nanosecond timescale such that the spectrum of the NIR pulse is retrieved measuring the temporal intensity trace using a fast photodiode (12 GHz, 32 ps rise time) and oscilloscope (Tektronix MSO68B, 10 GHz bandwidth, 25 GSamples/s). Overall, this scheme measures the THz waveform quite accurately. The standard deviation over 10 measurements typically corresponds to 10% but can still be improved by removing external sources of noise due to air current and vibrations.

2. Derivation of $\boldsymbol{E_{THz} \propto \sqrt{S_{THz}} - \sqrt{S_{ref}}}$

Using Jones matrices, the THz detection conditions used in the main manuscript are derived. The birefringent crystal used for detection is represented as

$$C = \begin{pmatrix} e^{-\frac{in_sL}{\lambda}} & 0 \\ 0 & e^{-\frac{in_fL}{\lambda}} \end{pmatrix} = e^{-i\Theta} \begin{pmatrix} e^{-\frac{i\Gamma}{2}} & 0 \\ 0 & e^{\frac{i\Gamma}{2}} \end{pmatrix}, \tag{S2.1}$$

where L is the crystal length, n_S and n_f are the indices of the slow and fast axes, respectively, and λ is the wavelength. With $\Theta = \frac{(n_s+n_f)L}{2\lambda}$, $\Gamma = \frac{(n_s-n_f)L}{\lambda} \propto \chi^{(2)} E_{THz}$ is the phase retardation experienced by the NIR pulse in the presence of a THz field due to the Pockels effect. Polarization optics include a quarter-wave plate (QWP) with its fast axis aligned horizontally

$$QWP = \begin{pmatrix} e^{-\frac{i\pi}{4}} & 0 \\ 0 & e^{\frac{i\pi}{4}} \end{pmatrix}, \tag{S2.2}$$

and polarizer whose T-axis is oriented at some angle β from the polarization of the NIR detection pulse (horizontal)

$$P(\beta) = (\cos\beta \quad \sin\beta). \tag{S2.3}$$

In our case, the linear polarization of the NIR detection pulse E_i is horizontal such that

$$E_i = E_0 \begin{pmatrix} 1 \\ 0 \end{pmatrix}. \tag{S2.4}$$

As a result, the electric field at the output of our detection scheme becomes

$$E_{out} = P(\beta)\ QWP\ R(-\alpha)\ C\ R(\alpha)\ E_i, \tag{S2.5}$$

where the rotation matrix

$$R(\alpha) = \begin{pmatrix} \cos\alpha & \sin\alpha \\ -\sin\alpha & \cos\alpha \end{pmatrix}, \tag{S2.6}$$

with $\alpha = \pi/4$ ensures maximum modulation from the THz field while maintaining phase information. The intensity $|E_{out}|^2$ yields the measured signal on the diode

$$S = E_0^2 \sin^2\left(\beta - \frac{\Gamma}{2}\right) \tag{S2.7}$$

$$S \approx E_0^2 \left(\beta - \frac{\Gamma}{2}\right)^2 \text{ for } \left|\beta - \frac{\Gamma}{2}\right| \ll 1. \tag{S2.8}$$

In our experiment, $\beta = 0.17$ rad and $\Gamma/2$ is always lower than 0.9 rad, ensuring that the condition defined in Eq. S2.8 is valid and we remain in a linear regime. Thus, without the THz field, the recorded reference signal becomes

$$S_{ref} = S(\Gamma = 0) = \beta^2. \tag{S2.9}$$

With the THz field inducing a birefringence on the crystal, we substitute Eq. S2.10 into the situation where $\Gamma \neq 0$.

$$S_{THz} = S(\Gamma \neq 0) \propto \left(\beta - \frac{\Gamma}{2}\right)^2 \tag{S2.10}$$

$$\sqrt{S_{THz}} \propto \beta - \frac{\Gamma}{2} = \sqrt{S_{ref}} - \frac{\Gamma}{2} \tag{S2.11}$$

$$\Gamma \propto \sqrt{S_{ref}} - \sqrt{S_{THz}}\ . \tag{S2.12}$$

Since the sign of the time domain signal is irrelevant and $E_{THz} \propto \Gamma$, we obtain the equation shown in Fig. 1d of the main manuscript.

$$E_{THz} \propto \sqrt{S_{ref}} - \sqrt{S_{THz}}. \tag{S2.13}$$

Here, the proportionality relationship, instead of an equality, is sufficient as we rely on normalized spectral components to calculate changes in the complex dielectric function of a material.

3. Multilayer method for evaluation of THz transmission

The transmission of the THz field through a photoexcited semiconductor is related to the dynamics and the transport of photocarriers. In bulk Si, photocarrier transport is well described by the Drude model. Moreover, the electron mobility is three times larger than the hole mobility; therefore it is assumed that charge transport is dominated by electrons[4]. Based on this assumption, the dielectric constant of Si, $\epsilon(\omega, z, t)$, can be expressed as:

$$\epsilon(\omega, z, t) = \epsilon_r + \frac{i}{\omega\epsilon_0}\sigma(\omega, z, t) = \epsilon_r + \frac{i}{\omega\epsilon_0}\frac{N(z,t)e^2\tau_s\big(N(z,t)\big)}{m^*\Big(1 - i\omega\tau_s\big(N(z,t)\big)\Big)}, \tag{S3.1}$$

where ω is the THz angular frequency, z is the distance along the THz field propagation, t is time, $\epsilon_r = 11.72$ is the relative real part of the permittivity for Si at room temperature[5], $\epsilon_0 = 8.85 \times 10^{-12}$ F m^{-1} is the free-space permittivity, $\sigma(\omega, z, t)$ is the complex conductivity, $m^* = 0.2 \times 9.11 \times 10^{-31}$ kg is the electron effective mass[4], e is the elementary charge, and $N(z, t)$ is the carrier density. The carrier density-dependent scattering time, $\tau_s\big(N(z,t)\big)$, is expressed as:

$$\tau_s = \tau_s\big(N(z,t)\big) = \tau_0\left(1 + \sqrt{\frac{N(z,t)}{N_{ref}}}\right)^{-1}, \tag{S3.2}$$

where $\tau_0 = 190$ fs is the initial scattering time, and phenomenological parameter $N_{ref} = 10^{17}$ cm^{-3} [4]. The corresponding THz power absorption coefficient can be expressed as:

$$\alpha_{THz}(\omega, z, t) = \frac{2\omega}{c} Im\left(\sqrt{\epsilon(\omega, z, t)}\right), \tag{S3.3}$$

where c is the speed of light in vacuum. For a uniformly excited sample, the THz field transmission $T(\omega, t)$ can be expressed using the Beer-Lambert law as[6]:

$$T(\omega, z, t) = e^{-\alpha_{THz}(\omega,z,t)d/2}. \tag{S3.4}$$

The spatial distribution of the photocarriers is not uniform upon injection as the optical pump power decreases exponentially within the sample and the photocarrier distribution is expected to change at early times after the injection due to diffusion. The multilayer method has been proven to be an accurate method to evaluate the transmission over non-uniformly distributed carriers in photoexcited samples[7]. This approach considers the photoexcited sample to consist of several layers of identical thickness where the dielectric permittivity $\epsilon(\omega, z, t)$ is constant over the considered layer. Figure S2 illustrates the partition of the photocarrier distribution into several layers. The photocarrier density in the pth layer is

$$N(z = pd, t = 0) = N_0 e^{-(p-1)\alpha_{pump}d} \approx N_0 e^{-\alpha_{pump}z}, \tag{S3.5}$$

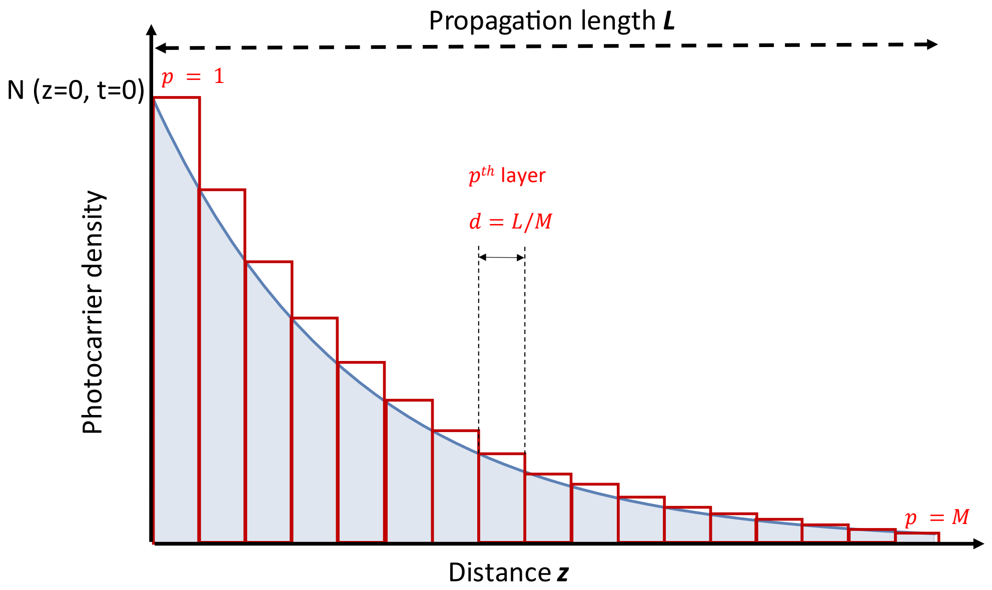

Figure S2. Spatial distribution of carriers upon optical injection. The pump propagation distance is divided in M layers of equal thickness d where permittivity is assumed constant.

where $\alpha_{pump} = 30.2$ cm^{-1} for our pump wavelength of 1030 nm[8], and $d = L/M$ is the thickness of a single layer. L is the total length of the sample and M is the total number of layers considered. The total THz field transmission over all layers can be expressed as the product of the THz transmission over all layers

$$T_{total}(\omega, z, t) = \prod_{p=1}^{M} e^{-\alpha_{THz}^{(p)}(\omega,z,t)d/2}, \quad \text{(S3.6)}$$

where $\alpha_{THz}^{(p)}(\omega, z, t)$ is the THz absorption of the pth layer. The scattering time τ_s is assumed to be carrier density-dependent following Eq. 3.2. In this work, the value $M = 60$ was used for all simulations and corresponds to $d = 15$ µm. The derived expression of the transmission in Eq. 3.6 is used to evaluate the time-dependent transmission once the photocarrier dynamics over each layer are obtained.

3.1. Photocarrier dynamics and spatial distribution

The equation governing free- and trapped-carrier dynamics is given by the carrier rate equation in one-dimension expressed as[9–12]:

$$\frac{dN(z,t)}{dt} = G(t) + D(N(z,t))\frac{d^2N(z,t)}{dz^2} - \frac{N(z,t)}{\tau_{eff}(t)} \quad \text{(S3.7)}$$

$$\frac{dN_{trap}(z,t)}{dt} = \frac{-N_{trap}(z,t) + N(z,t)}{\tau_{eff}(t)} \quad \text{(S3.8)}$$

where $N(z,t)$ is the density of free carriers, $G(t)$ is a photocarrier source function that generates photocarriers into the system at a rate of 50 kHz, $D(N(z,t))$ is the carrier density-dependent diffusion coefficient, $\tau_{eff}(t)$ is the trapped carrier density-dependent recombination time[12], and $N_{trap}(z,t)$ is the density of trapped photocarriers[13]. The trap-assisted recombination time is of the form:

$$\tau_{eff}(t) = \tau_R / \left(1 - \frac{N_{trap}(z,t)}{N_{trap}^{max}}\right), \quad \text{(S3.9)}$$

where $\tau_R = 1/(\tau_{SRH}^{-1} + \tau_{rad}^{-1} + \tau_{Auger}^{-1})$ considers Shockley-Read-Hall (τ_{SRH}), radiative (τ_{rad}), and Auger (τ_{Auger}) recombination times [12], and N_{trap}^{max} is the density of available traps[13]. Equations 7 and 8 assume a uniform distribution of photocarriers in the plane of incidence of the pump beam. At early times after injection, the spatial distribution of photocarriers is expected to follow the exponential decay $N(z,t) = N(0,t)e^{-\alpha_{pump}z}$ assuming every pump photon generates an electron-hole pair. This inhomogeneous distribution is important in evaluating the total THz transmission as the penetration depth of the pump pulse $\alpha_{pump}^{-1} = 0.33$ mm is small compared to the optical propagation length of 0.9 mm. Moreover, the Si sample is periodically injected every 20 µs. By considering that photocarrier recombination is larger than the pump period, the diffusion length $L_D = \sqrt{D\tau_{decay}} = 0.26$ mm is smaller than the penetration depth. This condition entails two important consequences: the first is that of a significant impact of diffusion in the overall photocarrier dynamics, and hence on the accumulation of carriers upon successive injection, and the second implication is related to a modification of the spatial distribution of photocarriers with time as carriers diffuse in all directions. In both instances, solving Eq. 3.7 requires an accurate description of the

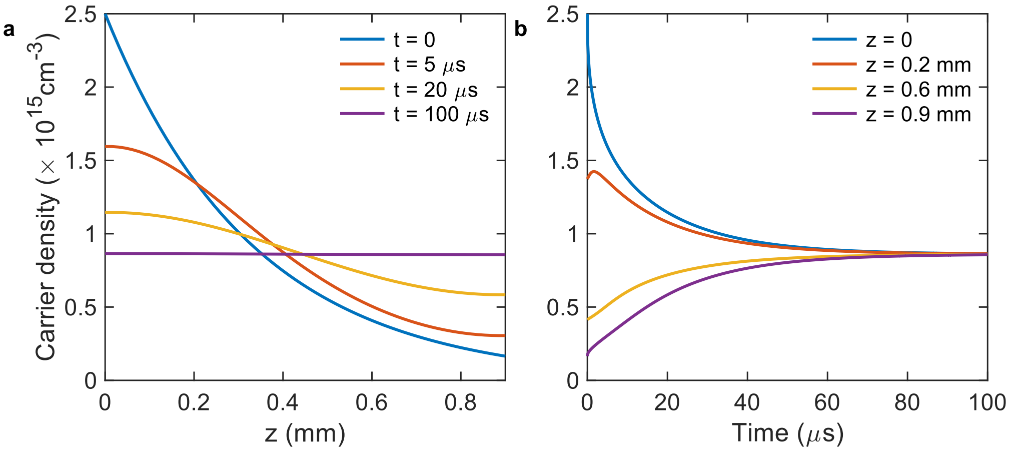

Figure S3. Time-varying carrier distribution with diffusion only. (a) Spatial distribution of photocarrier density at different points in time, (b) photocarrier dynamics at different points in space. Parameters considered are $N_d(z = 0, t = 0) = 2.5 \times 10^{15}$ cm^3, $D = 38$ cm^2/s, and $\alpha_{pump} = 30.2$ cm^{-1}.

spatial distribution of photocarriers at all times t. Previous pump-probe experiments address this issue by considering that photocarriers keep an exponential decay distribution over the probing duration[10,14]. This approximation is valid when the probing duration (a few hundred picoseconds) is very small compared to the recombination time. However, in the present case of bulk Si, the microsecond to millisecond time scale of carrier dynamics is of the same order of magnitude as the recombination time in bulk Si, and hence strengthens the need for proper modeling of $N(z, t > 0)$.

From the exponential distribution at $t = 0$, we approximate the second derivative in the diffusion term in Eq. 3.7 at a given point in space (z_i) and time (t_j) with the help of central difference approximations from neighboring carrier densities ($N(z_{i+1}, t_j)$ and $N(z_{i-1}, t_j)$).

$$\frac{d^2N(z,t)}{dz^2} = \frac{N(z_{i+1},t_j) + N(z_{i-1},t_j) - 2N(z_i,t_j)}{dz^2}. \tag{S3.10}$$

Figure S3a illustrates the spatial distribution of photocarriers following an injection of 2.5×10^{15} cm^{-3} carriers when calculating diffusion effects in this manner. The diffusion coefficient $D = 38$ cm^2 s^{-1} (for initial scattering time $\tau_0 = 190$ fs at room temperature[4]) is kept fixed in Fig. S3a, but in reality is proportional to the carrier-density dependent scattering time described in Eq. 3.2. The calculation results found in Fig. 3 of the main manuscript and Fig. S4 utilize this carrier dependent diffusion coefficient $D(N(z,t)) = \tau_s(N(z,t))k_B T/m^*$, where k_B is the Boltzmann constant and $T = 300$ K. At $t = 0$, the photocarrier distribution corresponds to an exponential decay as expected from the pump energy absorption in the sample. As time increases, this spatial distribution evolves from an exponential decay towards a uniform distribution over the entire sample thickness. Figure S3b shows the corresponding photocarrier dynamics resulting from diffusion alone at various points in space throughout the 0.9 mm thick wafer. It can be seen that carrier density at the front interface ($z = 0$) decreases rapidly shortly after excitation. On the other hand, the photocarrier density at the rear interface increases with time due to carriers diffusing from the front interface. The dynamics reach equilibrium at ~100 µs where carriers are uniformly distributed, and their density remains constant with time.

3.2. Photocarrier dynamics and time-varying transmission

To evaluate the carrier dynamics given by Eqs. 7 and 8 over time, we use a split-step method between carrier diffusion and recombination over time steps of 5 ns and spatial steps of 15 µm. For each point in time and space, the carrier-dependent dielectric function (Eq. 3.1), total THz transmittance (Eq. 3.6), and recombination time (Eq. 3.9) are calculated. The input parameters for the simulation results in Fig. 3 of the

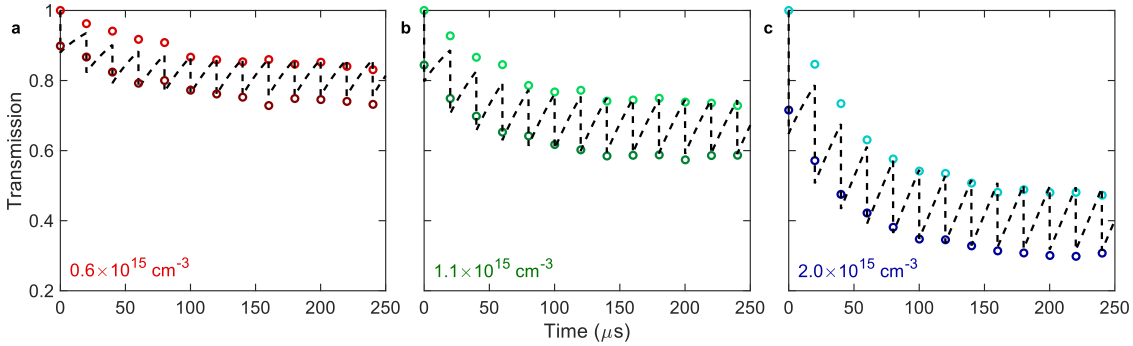

Figure S4. Time-varying THz transmission. The peak of the THz transient as the pump pulses arrive before (dark circles) and after (light circles) the THz pulse for carrier densities of a) 0.6, b) 1.1, and c) 2×10^{15} cm^{-3}. The black dashed lines represent the results from our theoretical analysis at $\omega = 0.9$ THz with the input parameters $\tau_R = 30$ µs, $N_{trap}^{max} = 6\times10^{11}$ cm^{-3}.

main manuscript are $\omega = 0.9$ THz (peak THz frequency in our experiments), $\tau_R = 30$ µs, $N_{trap}^{max} = 6\times10^{11}$ cm^{-3}. We use N_0, τ_R, and N_{trap}^{max} as fitting parameters for the experimental data. Low-doped silicon has an initial recombination time τ_R on the order of tens of microseconds[12], and the number of available traps should be much less than the injected number of carriers in a single optical pulse[13]. The values we have used for our simulations are well within these constraints.

Here, $G(t)$ injects the carrier distribution described in Eq. 3.5 every 20 µs. This periodic pumping is relaxed by carrier recombination and the resulting dynamics are that of an accumulation of photocarriers in the conduction band over time. Figure S4 displays the corresponding THz transmission evaluated at the peak frequency of the THz field amplitude considering $M = 60$ layers (15 µm step size) for the three injected carrier densities considered in our experiments (Fig. 3 of the main manuscript). To calculate the theoretical THz amplitude transmission spectrum, our calculations are repeated for frequencies within the bandwidth of the THz pulse (inset Fig. 1d of the main manuscript). The numerical simulations are in good agreement with experimental results.

References

1. Jiang, Z. & Zhang, X.-C. Electro-optic measurement of THz field pulses with a chirped optical beam. *Appl. Phys. Lett.* **72**, 1945–1947 (1998).
2. Hebling, J., Yeh, K.-L., Hoffmann, M. C., Bartal, B. & Nelson, K. A. Generation of high-power terahertz pulses by tilted-pulse-front excitation and their application possibilities. *JOSA B* **25**, B6–B19 (2008).
3. Blanchard, F. *et al.* Generation of 1.5 µJ single-cycle terahertz pulses by optical rectification from a large aperture ZnTe crystal. *Opt. Express* **15**, 13212–13220 (2007).
4. Jacoboni, C., Canali, C., Ottaviani, G. & Alberigi Quaranta, A. A review of some charge transport properties of silicon. *Solid-State Electron.* **20**, 77–89 (1977).
5. Krupka, J. *et al.* Measurements of Permittivity, Dielectric Loss Tangent, and Resistivity of Float-Zone Silicon at Microwave Frequencies. *IEEE Trans. Microw. Theory Tech.* **54**, 3995–4001 (2006).
6. Andorn, M. & Bar-Eli, K. H. Optical Bleaching and Deviations from Beer—Lambert's Law of Solutions Illuminated by a Ruby Laser. II. Sodium—Propylenediamine Solutions. *J. Chem. Phys.* **55**, 5017–5020 (1971).
7. Matsuzaki, H. *et al.* Photocarrier dynamics in anatase TiO2 investigated by pump-probe absorption spectroscopy. *J. Appl. Phys.* **115**, 053514 (2014).
8. Green, M. A. Self-consistent optical parameters of intrinsic silicon at 300K including temperature coefficients. *Sol. Energy Mater. Sol. Cells* **92**, 1305–1310 (2008).
9. *Semiconductor Physical Electronics*. (Springer, 2006). doi:10.1007/0-387-37766-2.
10. Zakar, A. *et al.* Carrier dynamics and surface vibration-assisted Auger recombination in porous silicon. *Phys. Rev. B* **97**, 155203 (2018).
11. He, W., Wu, R., Yurkevich, I. V., Canham, L. T. & Kaplan, A. Reconstructing charge-carrier dynamics in porous silicon membranes from time-resolved interferometric measurements. *Sci. Rep.* **8**, 17172 (2018).
12. Schroder, D. K. Carrier lifetimes in silicon. *IEEE Trans. Electron Devices* **44**, 160–170 (1997).
13. Yokoyama, K., Lord, J. S., Miao, J., Murahari, P. & Drew, A. J. Photoexcited Muon Spin Spectroscopy: A New Method for Measuring Excess Carrier Lifetime in Bulk Silicon. *Phys. Rev. Lett.* **119**, 226601 (2017).
14. Němec, H. *et al.* Ultrafast carrier dynamics in Br+-bombarded InP studied by time-resolved terahertz spectroscopy. *Phys. Rev. B* **78**, 235206 (2008).
